# Identifying putative causal links between serum circulating microRNAs and thyroid cancer using Mendelian randomization

**DOI:** 10.1007/s12672-025-04125-3

**Published:** 2025-12-08

**Authors:** Xiaojin Fu

**Affiliations:** Zhejiang Sian International Hospital, Jiaxing, 314000 Zhejiang China

**Keywords:** Thyroid cancer, microRNAs, Mendelian randomization, Biomarkers, Risk prediction

## Abstract

**Background:**

Thyroid cancer, the most common malignancy in the endocrine system, has seen a global increase in incidence. This study aims to investigate the causal relationship between serum circulating microRNAs (miRNAs) and thyroid cancer risk using a Mendelian randomization (MR) approach.

**Methods:**

We conducted a two-sample MR analysis using miRNA expression quantitative trait loci (eQTL) data from two independent cohorts and thyroid cancer genome-wide association study (GWAS) data. The discovery cohort included miRNA expression levels quantified via qRT-PCR in whole blood samples, while the validation cohort comprised miRNA expression data from blood samples of unrelated individuals of European ancestry. We applied various MR methods, including inverse variance-weighted (IVW) and MR-Egger, to assess the genetic associations between miRNAs and thyroid cancer. Additionally, we performed target gene prediction and pathway analysis to explore the biological mechanisms underlying the observed associations.

**Results:**

Our analysis identified three miRNAs significantly associated with thyroid cancer risk: miR-hsa-125b-5p and miR-hsa-30a-3p were found to have harmful effects, while miR-hsa-130a-3p exhibited a protective effect. These findings were consistently validated across both cohorts. The target genes of these miRNAs were enriched in pathways related to gland development, myeloid cell differentiation, cellular senescence, FoxO signaling pathway, and p53 signaling pathway, providing insights into the potential molecular mechanisms linking these miRNAs to thyroid cancer.

**Conclusion:**

This study suggests potential causal associations between specific serum circulating miRNAs and thyroid cancer risk using a Mendelian randomization approach. The identified miRNAs, including miR-hsa-125b-5p, miR-hsa-30a-3p, and miR-hsa-130a-3p, could be further investigated as potential biomarkers for risk prediction and early diagnosis of thyroid cancer.

**Supplementary Information:**

The online version contains supplementary material available at 10.1007/s12672-025-04125-3.

## Introduction

 Thyroid cancer is one of the most common malignant tumors in the endocrine system. According to GLOBOCAN 2022 data, the global annual incidence rate of thyroid cancer grows by approximately 3%, with some high-income countries experiencing an increase as high as 5% [1]. Thyroid cancers can be primarily categorized into papillary carcinoma, follicular carcinoma, medullary carcinoma, and undifferentiated carcinoma based on their different pathological types. Among these, papillary carcinoma is the most common type, accounting for about 70%−80% of all thyroid cancer cases [2]. Papillary carcinoma has a relatively good prognosis, which is mainly attributed to its slow growth and low local invasiveness. In contrast, undifferentiated carcinoma exhibits highly aggressive behavior and has a very poor prognosis [2]. Although most patients with thyroid cancer have favorable outcomes after early detection and treatment, a portion of patients, especially those with high-risk factors (such as older age, larger tumors, distant metastasis), face higher risks of recurrence and poorer survival rates [3]. Therefore, identifying new biomarkers is of significant importance for improving risk prediction, early diagnosis, and the development of treatment strategies for thyroid cancer.

MicroRNAs (miRNAs) are a class of approximately 22-nucleotide non-coding RNA molecules that play indispensable regulatory roles in various physiological processes such as cell differentiation, proliferation, and apoptosis. For instance, during heart development, specific miRNAs ensure normal cardiac development by regulating the expression of genes related to cardiomyocytes [4]. Pathologically, miRNAs are also involved in the onset and progression of numerous diseases, including cancer [5]. In recent years, extensive research has focused on the relationship between serum circulating miRNAs and thyroid cancer, revealing not only their involvement in the development and progression of thyroid cancer but also their potential as biomarkers for disease diagnosis and prognosis evaluation [6, 7]. Certain specific miRNAs exhibit significantly different expression levels in thyroid cancer tissues compared to normal tissues, and these differentially expressed miRNAs can be detected in the bloodstream, suggesting their potential as ideal candidates for non-invasive diagnostic and monitoring tools [8]. Furthermore, miRNAs may influence tumor malignancy and prognosis by regulating biological behaviors such as the proliferation, migration, and invasion of thyroid cancer cells [9]. Additionally, miRNAs hold potential application value in the treatment of thyroid cancer, such as achieving targeted therapy through modulating miRNA expression levels [10]. However, due to the influences of various confounding factors like environmental factors and lifestyle, studies directly linking miRNAs to thyroid cancer risk often suffer from biases, limiting their promotion in clinical applications.

To overcome the limitations of confounding bias and reverse causation inherent in traditional observational studies, this study employs a two-sample Mendelian Randomization (MR) approach to systematically assess the causal relationship between serum circulating miRNAs and thyroid cancer risk for the first time. Utilizing large-scale miRNA expression quantitative trait loci (eQTLs) data and international thyroid cancer genome-wide association study (GWAS) consortium data, we validate our findings using multiple methods, including inverse variance weighted (IVW) and MR-Egger regression, while further exploring the target genes and potential pathways regulated by significant miRNAs through enrichment analysis. The aim of this study is to provide high-evidence-grade molecular markers for the early, non-invasive diagnosis of thyroid cancer.

## Materials and methods

### Acquisition of miRNA eQTL data

To explore the genetic regulation of miRNAs in relation to thyroid cancer, we sourced data from two previous studies. The discovery cohort was derived from research conducted by Huan et al. [11], where miRNA expression levels were quantified via quantitative reverse transcription PCR (qRT-PCR) in whole blood samples from participants. This dataset initially contained 280 high-quality miRNAs associated with approximately 10 million SNPs. Our analysis focused solely on cis-acting miRNA-eQTLs, with further exclusion of SNPs located within coding regions that could induce synonymous or missense mutations, aiming to minimize potential pleiotropy. Following these criteria, we selected SNPs as instrumental variables based on a Benjamini-Hochberg corrected false discovery rate (FDR) < 0.1. Additionally, validation cohort data were obtained from Nikpay et al.‘s study [12], which included miRNA expression levels from blood samples of 710 unrelated individuals of European ancestry. MiRNA IDs across both datasets were standardized using miRCarta v1.1.

### Thyroid cancer GWAS data collection

Genome-wide association study (GWAS) data for thyroid cancer were retrieved from the IEU OpenGWAS Project (accessed December 12, 2024), under GWAS ID ebi-a-GCST90018929. This dataset encompasses 1,054 cases and 490,920 controls, all of European descent, totaling 24,198,226 SNPs.

### Mendelian randomization analysis

Two-sample Mendelian randomization (MR) analyses were performed using the R package TwoSampleMR version 0.6.8. MR-Egger and inverse variance-weighted (IVW) methods were utilized to assess the genetic associations between miRNAs and thyroid cancer. Initially, independent instrumental variables (IVs) were identified through linkage disequilibrium clumping with an r² threshold of < 0.5 and a 10 kb window. We then extracted SNPs from the thyroid cancer GWAS data that showed no significant association with breast cancer (*p* > 5e-08). Both exposure and outcome datasets were harmonized to ensure common effect alleles, with palindromic SNPs being excluded. For a miRNA to be considered causally linked to thyroid cancer, it had to meet the following criteria: (1) IVW test p-value < 0.05 and FDR < 0.1; (2) consistent direction of effect sizes across both IVW and MR-Egger models; (3) inclusion of at least three SNPs in the MR tests; and (4) confirmation of significant results in the validation cohort’s IVW analysis. Potential horizontal pleiotropy was evaluated using MR-Egger regression, while leave-one-out analysis was applied to examine the stability of causal estimates by sequentially excluding individual SNPs.

### Target gene prediction and pathway analysis

Target genes for the identified miRNAs were predicted using miRNet 2.0, focusing exclusively on experimentally validated targets as documented in the miRTarBase v9.0 database. These miRNA-target interactions were visualized as networks using Cytoscape v3.10.2. Functional annotations, including Gene Ontology (GO) enrichment and KEGG pathway analysis, were performed using the ClusterProfiler v4.12.6 package, with significance defined by a false discovery rate (FDR) < 0.05.

## Results

### Characteristics of serum miRNA eQTL data

**Fig. 1:**
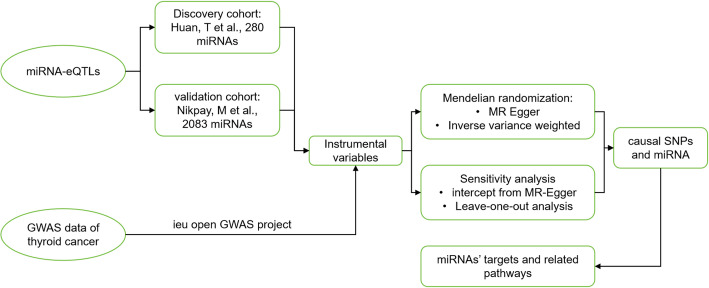
An overview of the study workflow

Initially, we conducted preprocessing on the cohort eQTL data from Huan et al., which included filtering out 9,517 SNPs that did not have a direct association with coding genes from an initial set of 9,612 single nucleotide polymorphisms (SNPs). Additionally, 17 SNPs related to mir-213 were excluded due to their inability to map to valid miRNA IDs. This rigorous selection process resulted in a final dataset consisting of 9,500 SNPs serving as instrumental variables for 75 miRNAs suitable for Mendelian Randomization (MR) analysis (Table S1). To further validate our findings, we utilized data from Nikpay et al.‘s study, involving 164,336 SNPs associated with 2,083 miRNAs (*p* < 1e-5). An overview of the study workflow is presented in Fig. 1.

### Beneficial and harmful effects of serum circulating miRNAs on thyroid cancer

**Fig. 2:**
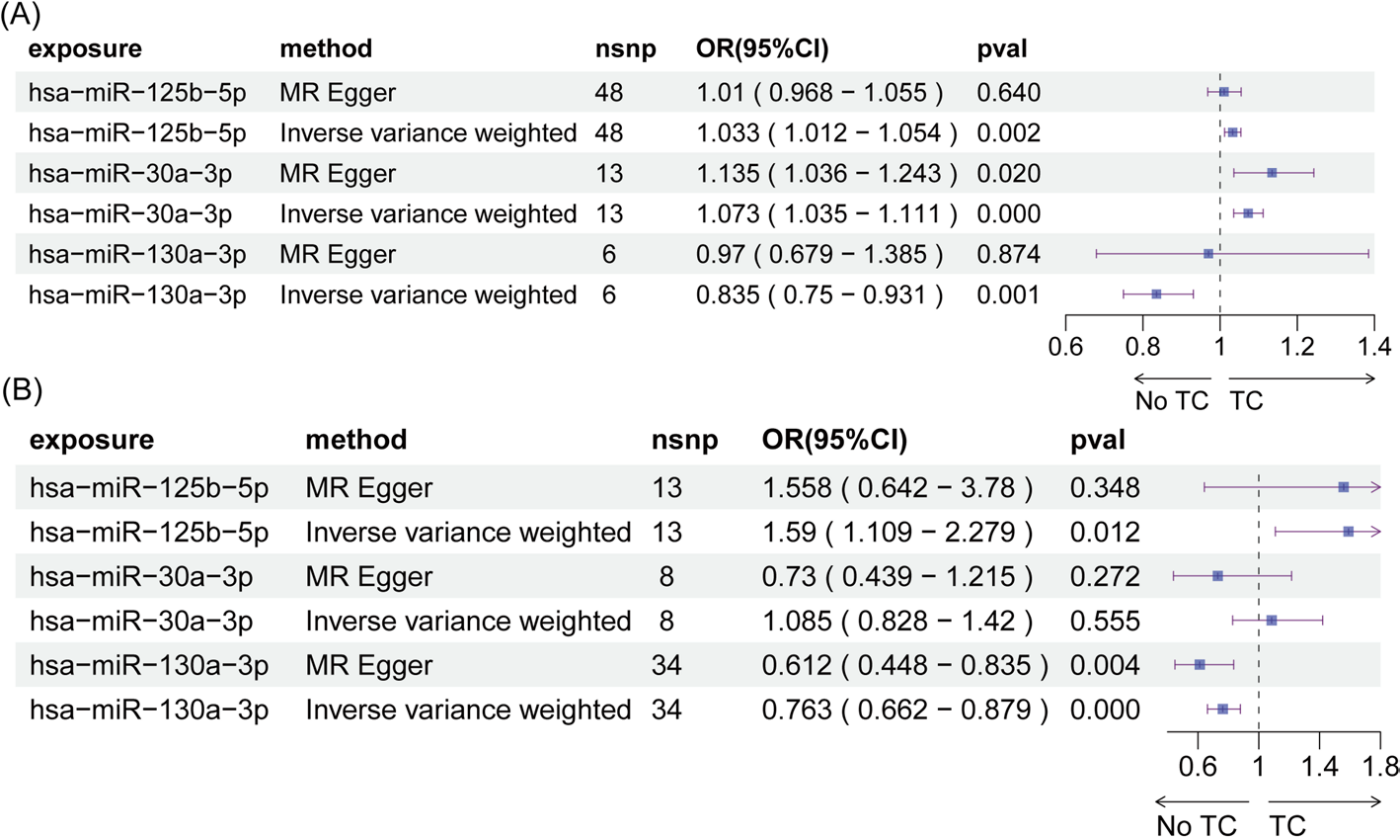
Association of serum circulating miRNAs with thyroid cancer. **A** Association of serum circulating miRNAs with thyroid cancer in the Huan et al. cohort. **B** Validation of the three causal miRNAs in relation to thyroid cancer in the Nikpay et al. cohort

The relationship between serum circulating miRNAs and thyroid cancer was investigated within the cohort by Huan et al., as shown in Fig. 2A. Our MR analysis indicated that miR-hsa-125b-5p and miR-hsa-30a-3p were associated with an increased risk of thyroid cancer, while miR-hsa-130a-3p was associated with a decreased risk. For validation purposes, the three causal miRNAs—miR-hsa-125b-5p, hsa-miR-30a-3p, and hsa-miR-130a-3p—were assessed in the cohort by Nikpay et al. (Fig. 2B). The results further confirmed the detrimental effect of miR-hsa-125b-5p and the beneficial effect of hsa-miR-130a-3p on thyroid cancer. These analyses substantiate the importance of these miRNAs in influencing thyroid cancer risk.

### Pleiotropy tests and sensitivity analysis

**Fig. 3:**
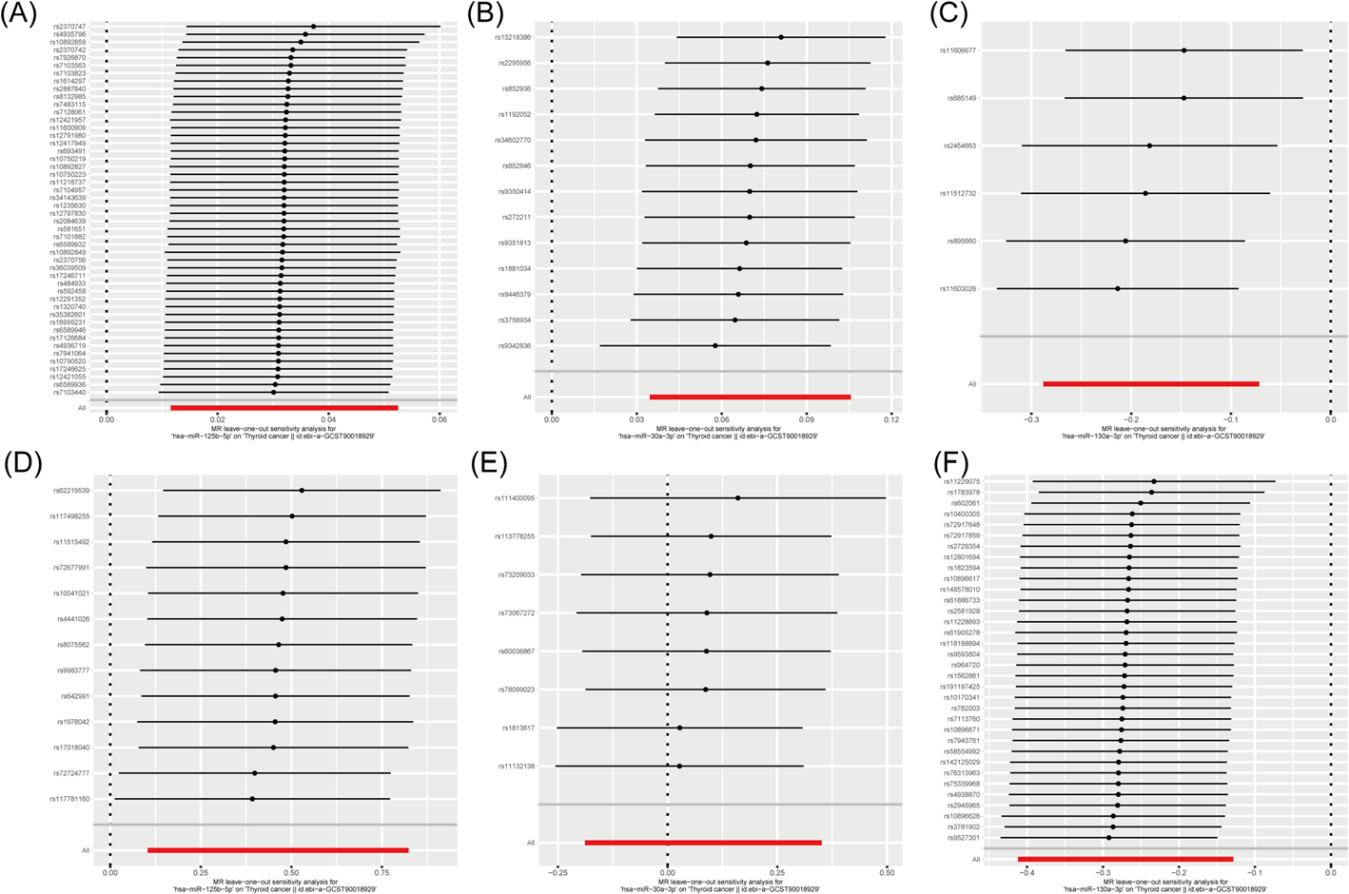
Results from Leave-One-Out Sensitivity Analysis. Leave-one-out sensitivity analysis for instrumental variables in (**A**) hsa-miR-125b-5p, (**B**) hsa-miR-30a-3p, and (**C**) hsa-miR-130a-3p within the Huan et al. cohort. Similarly, leave-one-out sensitivity analysis for instrumental variables in (**D**) hsa-miR-125b-5p, (**E**) hsa-miR-30a-3p, and (**F**) hsa-miR-130a-3p within the Nikpay et al. cohort

Table 1 summarizes the results of pleiotropy tests for instrument effects across discovery and validation datasets. The Egger intercept values, standard errors, and p-values for hsa-miR-125b-5p, hsa-miR-30a-3p, and hsa-miR-130a-3p indicated the robustness of these miRNAs as reliable instruments. Further validation of these findings was achieved through leave-one-out sensitivity analyses, illustrated in Fig. 3A–F. These figures showed the stability and consistency of the effect estimates when individual SNPs are sequentially removed from the analysis, both in the Huan et al. and Nikpay et al. cohorts.

**Table 1:** Pleiotropy tests of instrument effects

Exposure	Discovery dataset			Validation dataset		
	Egger_intercept	se	pval	Egger_intercept	se	pval
hsa-miR-125b-5p	0.0181	0.0160	0.2641	0.0018	0.0373	0.9615
hsa-miR-30a-3p	−0.0463	0.0354	0.2178	0.0741	0.0413	0.1229
hsa-miR-130a-3p	−0.0544	0.0630	0.4368	0.0306	0.0195	0.1271

### Targets and pathways related to causal miRNAs

**Fig. 4:**
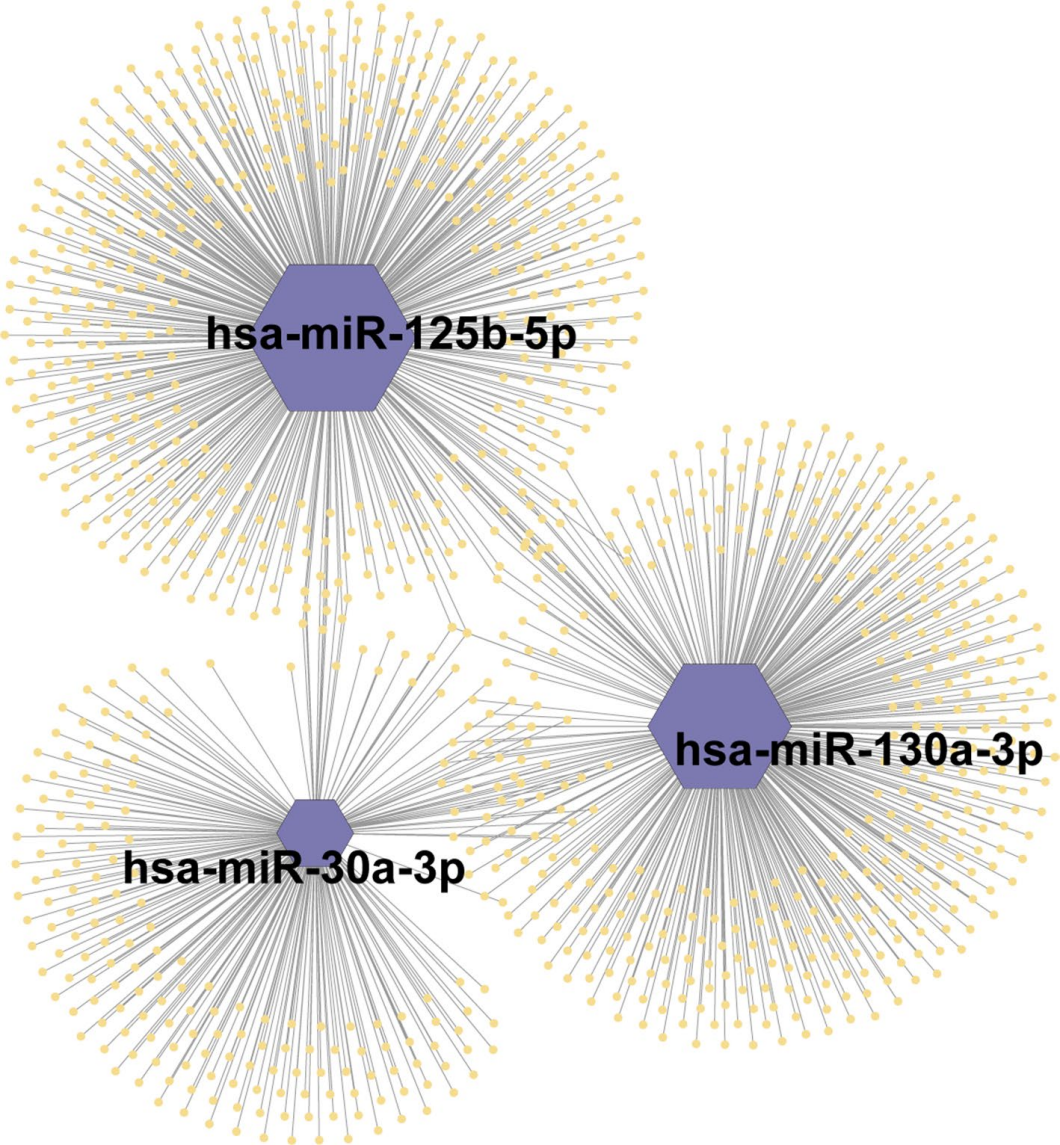
Network diagram of causal miRNAs and their targets. Purple nodes represent miRNAs, and yellow nodes denote corresponding targets

**Fig. 5:**
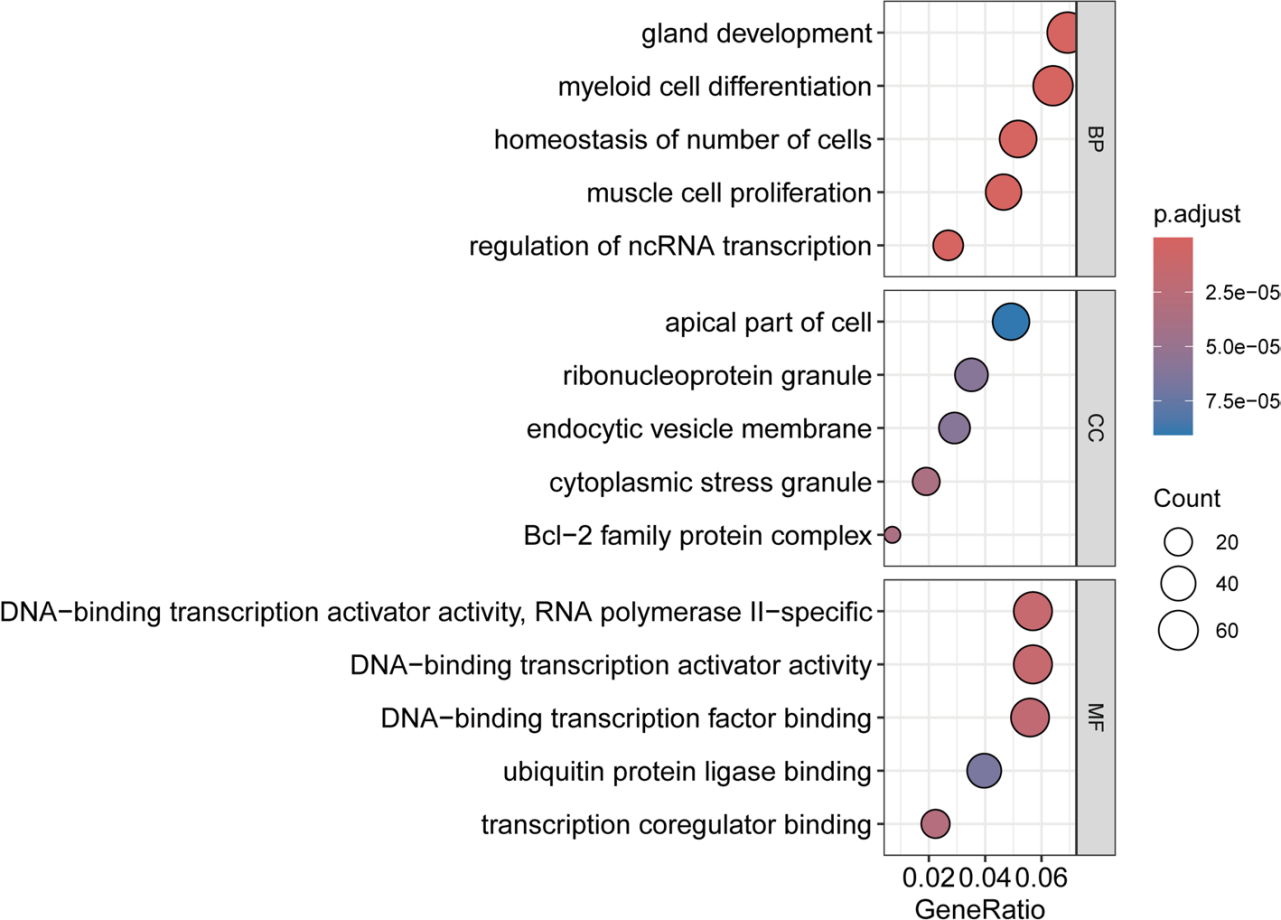
GO enrichment analysis of nodes related to causal miRNAs

**Fig. 6:**
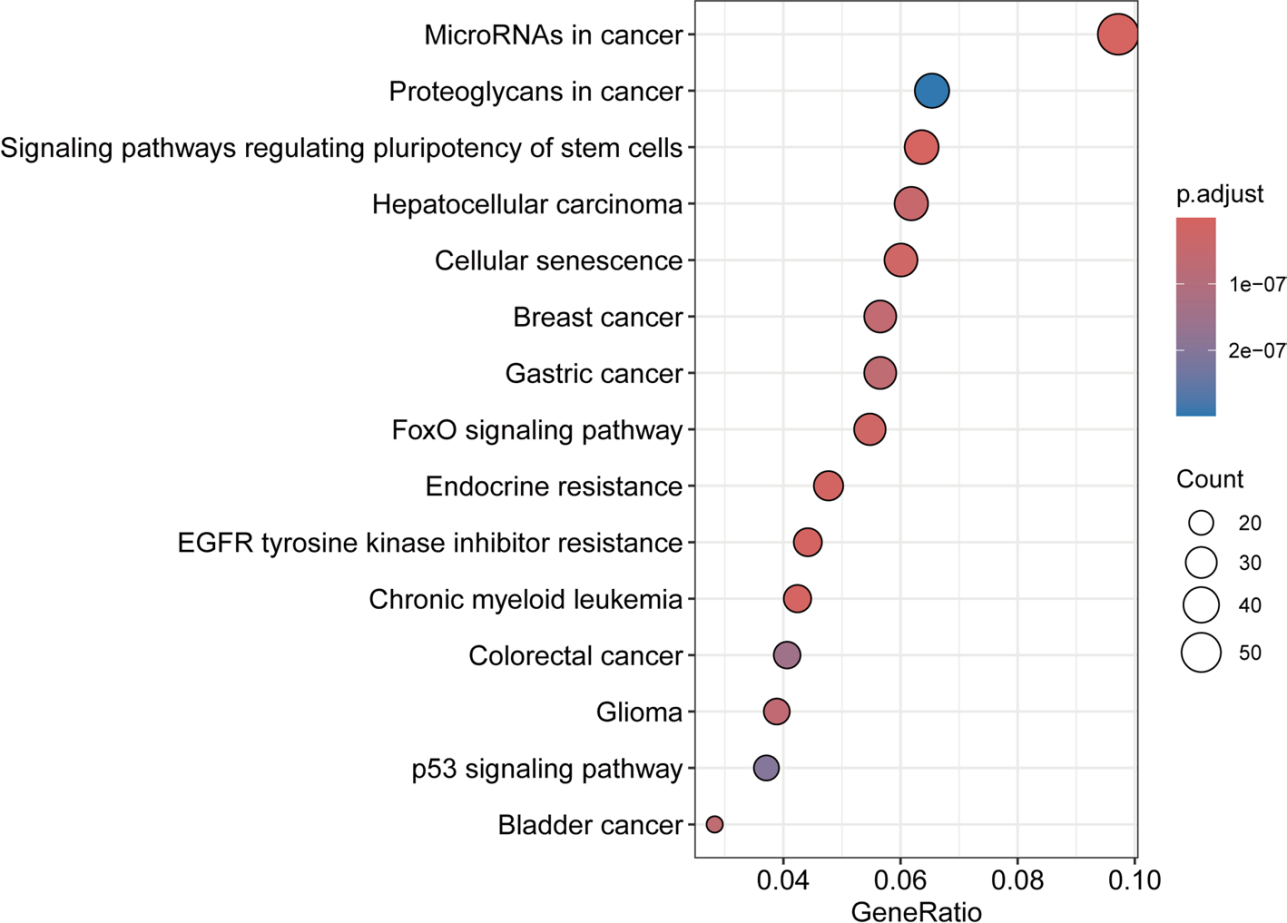
KEGG pathway enrichment analysis of nodes related to causal miRNAs

To elucidate the biological mechanisms underlying the observed associations, we constructed a network diagram illustrating the interactions between causal miRNAs and their predicted gene targets (Fig. 4, Table S2). In this network, purple nodes represent miRNAs, while yellow nodes indicate target genes. Specifically, hsa-miR-125b-5p has 494 targets, hsa-miR-130a-3p has 416 targets, and hsa-miR-30a-3p has 212 targets. Functional annotation of the causal miRNA-related nodes was performed using Gene Ontology (GO) enrichment analysis (Fig. 5, Table S3), revealing that these targets are associated with gland development, myeloid cell differentiation, transcription, and ubiquitination. Moreover, KEGG pathway enrichment analysis (Fig. 6, Table S4) identified critical pathways potentially influenced by these miRNAs, including cellular senescence, FoxO signaling pathway, p53 signaling pathway, among others, providing deeper insights into the molecular mechanisms linking circulating miRNAs to thyroid cancer.

## Discussion

This study systematically evaluated the causal relationship between serum circulating miRNAs and thyroid cancer risk using a two-samp le Mendelian Randomization (MR) approach. Our findings revealed significant associations between three specific miRNAs (miR-hsa-125b-5p, miR-hsa-30a-3p, and miR-hsa-130a-3p) and thyroid cancer risk. Among these, miR-hsa-125b-5p and miR-hsa-30a-3p exhibited harmful effects, whereas miR-hsa-130a-3p showed protective effects. These findings were not only validated in two independent cohorts but also further elucidated through target gene prediction and pathway analysis to reveal potential biological mechanisms.

Our findings hold significant promise for improving the clinical management of thyroid cancer. The identified circulating miRNAs—miR-hsa-125b-5p, miR-hsa-30a-3p, and miR-hsa-130a-3p—could serve as novel, non-invasive biomarkers for risk assessment and early detection. Their stability in serum makes them ideal candidates for liquid biopsy, potentially enhancing the diagnostic accuracy for thyroid nodules and reducing unnecessary invasive procedures. Furthermore, the causal roles of these miRNAs suggest their potential as therapeutic targets; for instance, miR-130a-3p mimics or inhibitors of the oncogenic miR-125b-5p/miR-30a-3p could be explored as RNA-based therapeutics to modulate tumor progression. The Mendelian randomization design provides robust genetic evidence supporting these miRNAs, strengthening their credibility for clinical translation.

MiR-hsa-125b-5p has been reported to promote tumor growth and metastasis in various cancers and is considered a potential marker for several types of cancer [13–15]. For instance, Huang et al. demonstrated that miR-125b-5p promotes ovarian cancer growth and metastasis by targeting CD147 [16]. Additionally, Guo et al. found that cancer-associated fibroblast-derived exosomal miR-125b-5p enhances pancreatic cancer cell proliferation, migration, and invasion by downregulating APC expression [14]. In our study, miR-hsa-125b-5p was significantly associated with increased thyroid cancer risk, consistent with previous studies, further supporting its role as an oncogenic factor. MiR-hsa-125b-5p affects cell proliferation, migration, and invasion by targeting multiple tumor suppressor genes such as CD147 and TPD52 [16, 17], and it participates in cancer progression by targeting STAT3 [18], BMPR1B [19], VEGFA [20], SphK1 [21], CDKN2A [22], and Sema4D [23].

In contrast, miR-hsa-30a-3p has been reported to have tumor-suppressive roles in various cancers. For example, miR-30a-3p inhibits hepatocellular carcinoma cell proliferation via the PI3K/AKT signaling pathway by targeting DNMT3a [24] and suppresses renal cell carcinoma invasion and metastasis by targeting ATG12 [25]. Hsa-miR-30a-3p overcomes acquired protective autophagy during chemotherapy, inhibiting bladder cancer growth and muscle invasion [26]. Moreover, miR-30a-3p acts as a promising suppressor gene by negatively regulating GAST expression [27] and targets ANLN in breast cancer [28]. The expression of miR-30a-3p inhibits small cell lung cancer cell proliferation, induces cell cycle arrest, and apoptosis, potentially through targeting DONSON [29], and regulates gastric cancer cell proliferation by targeting MAD2L1 [30]. These results are contrary to those of our study, suggesting a unique role of miR-hsa-30a-3p in thyroid cancer that warrants further experimental investigation.

Unlike the aforementioned two miRNAs, serum circulating levels of miR-hsa-130a-3p exert beneficial effects on thyroid cancer. However, previous studies have shown that miR-130a-3p promotes cancer cell proliferation and invasion. For instance, miR-130a-3p promotes cervical cancer cell proliferation and invasion by targeting estrogen receptor α and androgen receptor [31]. Exosomal miR-130a-3p confers cisplatin resistance in esophageal cancer by regulating ferroptosis through m6A RNA methylation of FSP1 mediated by METTL14 [32]. MiR-130a-3p regulates the TGF-β1/SMAD3 pathway by targeting Glucosaminyl N-acetyl transferase 4 (GCNT4), promoting gastric carcinogenesis [33]. MiR-130a-3p inhibits human breast cancer stem-like cell migration and invasion by regulating RAB5B [34]. MiR-130a-3p is downregulated in triple-negative breast cancer and blocks the Wnt signaling cascade by targeting key players [35], a finding also observed in colorectal cancer [36]. Exosomal miR-130a-3p promotes differentiated thyroid cancer progression by targeting insulin-like growth factor 1 [37]. Exosome-derived miR-hsa-130a-3p shows high diagnostic value in predicting recurrence in resectable non-small-cell lung cancer patients [35]. Therefore, the functional and mechanistic roles of miR-130a-3p in thyroid cancer require further experimental confirmation.

Through target gene prediction and pathway analysis, we found that the target genes of these miRNAs are enriched in several pathways closely related to cancer initiation and progression, including gland development, myeloid cell differentiation, cellular senescence, FoxO signaling pathway, and p53 signaling pathway. The FoxO signaling pathway plays a crucial role in cancer development and progression [38]. For instance, activation of the FoxO signaling pathway promotes ferroptosis, inhibiting laryngeal cancer cell proliferation, migration, and invasion [39]. The AKT/FOXO signaling pathway is vital for prostate cancer cell apoptosis and chemosensitivity [40]. The p53 signaling pathway is one of the most important tumor suppression pathways. Ding et al. demonstrated that lncRNA FEZF1-AS1 inhibits colorectal cancer development by regulating the p53 signaling pathway [41].

Despite its strengths, this study has several limitations that should be considered. Firstly, the MR design relies on key assumptions. While we performed pleiotropy tests and sensitivity analyses, the potential for horizontal pleiotropy cannot be entirely ruled out, which might bias the causal estimates. Secondly, the strength of the instrumental variables (IVs) was limited by the availability of miRNA eQTLs, and weak instruments might affect the precision and reliability of our findings. Thirdly, the study population primarily consisted of individuals of European descent, limiting the generalizability of our findings to other ethnic populations. Future research in diverse ancestries is needed. Fourthly, the bioinformatic predictions of miRNA target genes and pathways, while based on experimentally validated databases, require functional experimental validation to confirm their roles in thyroid carcinogenesis. Fourth, our analysis was necessarily limited to the broad phenotype of ‘thyroid cancer’ as defined by ICD-10 code C73. We recognize that important biological differences exist between histological subtypes such as papillary, follicular, and medullary thyroid carcinomas. While subtype-specific GWAS datasets are emerging, their current sample sizes remain insufficient for statistically robust Mendelian randomization analysis due to concerns about weak instrument bias and inadequate power. Our findings likely reflect aggregate risk patterns, potentially dominated by the most common subtype (papillary thyroid cancer). Future studies with larger, subtype-specific genetic data are needed to elucidate potential differential effects of miRNAs across the thyroid cancer spectrum. Lastly, our analysis focused on circulating miRNAs in serum; the expression and regulatory mechanisms of tissue-specific miRNAs might differ and warrant separate investigation.

While our MR analysis provides strong genetic evidence for causality, the translational potential of miR-125b-5p, miR-30a-3p, and miR-130a-3p as biomarkers requires further experimental and clinical validation. Future studies should focus on: (1) measuring the expression levels of these miRNAs in serum and matched tumor tissue from independent cohorts of thyroid cancer patients (e.g., using qRT-PCR); (2) correlating their expression levels with clinical-pathological features (e.g., cancer stage, subtype, and prognosis); and (3) investigating their functional roles in thyroid cancer pathogenesis through in vitro and in vivo models.

## Conclusion

In conclusion, our study provides genetic evidence supporting potential causal roles of specific serum circulating miRNAs and thyroid cancer risk. These findings suggest that miR-hsa-125b-5p, miR-hsa-30a-3p, and miR-hsa-130a-3p warrant further investigation as potential biomarkers for risk prediction and early diagnosis of thyroid cancer. Future studies are needed to validate these associations in diverse populations and elucidate the underlying biological mechanisms through experimental models.

## Supplementary Information

Supplementary Material 1: Table S1. SNPs instrumental variables for Mendelian randomization analysis.

Supplementary Material 2: Table S2. Comprehensive list of experimentally validated target genes for the three causal miRNAs.

Supplementary Material 3: Table S3. The enriched GO terms of experimentally validated target genes for the three causal miRNAs.

Supplementary Material 4: Table S4. The enriched KEGG terms of experimentally validated target genes for the three causal miRNAs.

## Data Availability

The miRNA eQTL data from the Huan et al. cohort were obtained from the Supplementary Information of the corresponding publication (available at: https://www.nature.com/articles/ncomms7601#Sec26). The miRNA eQTL data from the Nikpay et al. cohort can be obtained from the corresponding author of the original article upon reasonable request. The thyroid cancer genome-wide association study (GWAS) data were sourced from the IEU OpenGWAS database (GWAS ID: ebi-a-GCST90018929; available at: https://gwas.mrcieu.ac.uk/datasets/ebi-a-GCST90018929/).
